# Vascular Accesses in Cardiac Stimulation and Electrophysiology: An Italian Survey Promoted by AIAC (Italian Association of Arrhythmias and Cardiac Pacing)

**DOI:** 10.3390/biology11020265

**Published:** 2022-02-08

**Authors:** Matteo Ziacchi, Angelo Placci, Andrea Angeletti, Fabio Quartieri, Cristina Balla, Santo Virzi, Matteo Bertini, Roberto De Ponti, Mauro Biffi, Giuseppe Boriani, for AIAC Ricerca Investigators’ Network

**Affiliations:** 1 Department of Experimental, Institute of Cardiology, Diagnostic and Specialty Medicine, University of Bologna, S. Orsola-Malpighi University Hospital, 40138 Bologna, Italy; andrea.angeletti89@gmail.com (A.A.); mbiffi64@gmail.com (M.B.); 2 Institute of Cardiology, University Hospital of Parma, 41100 Parma, Italy; drplacci@gmail.com; 3 Institute of Cardiology, Santa Maria Nuova Hospital of Reggio Emilia, 42100 Reggio Emilia, Italy; fabio.quartieri@ausl.re.it; 4 Institute of Cardiology, Sant’Anna University Hospital of Ferrara, 44124 Ferrara, Italy; cristina.balla@unife.it (C.B.); matteo.bertini@unife.it (M.B.); 5 Department of Emergency, Division of Cardiology, SS.ma Annunziata Hospital, 44042 Cento, Italy; santo.virzi@gmail.com; 6 Department of Cardiology, University of Insubria, Circolo e Fondazione Macchi di Varese Hospital, 21100 Varese, Italy; roberto.deponti@uninsubria.it; 7 Department of Biomedical, Metabolic and Neural Sciences, Cardiology Division, University of Modena and Reggio Emilia, Policlinico Di Modena, 41121 Modena, Italy; giuseppe.boriani@unimore.it

**Keywords:** vascular access, CIED, electrophysiology, survey

## Abstract

**Simple Summary:**

Both cardiac implantable electronic device (CIED) implantations and electrophysiology procedures require vascular access to reach the heart through vessels. Different types of access carry different rates of complications. Safety and ease of vascular access are the main targets of physicians; in fact, each complication causes morbidity and raises costs. To avoid complications, the use of ultrasound-guided vessel puncture and closure devices is increasing in frequency. We conducted a survey in Italian centers to outline common practice; an uneven pattern of habits emerged. Hopefully, recently published scientific society consensus statements will lead to an improvement in physicians’ practice. The survey highlights that there is an unmet need for dedicated courses, particularly for ultrasound-guided vessel puncture.

**Abstract:**

Cardiac implantable electronic device (CIED) implants and electrophysiological procedures share a common step: vascular access. On behalf of the AIAC Ricerca Investigators’ Network, we conducted a survey to outline Italian common practice regarding vascular access in EP-lab. All Italian physicians with experience in CIED implantation and electrophysiology were invited to answer an online questionnaire (from May 2020 to November 2020) featuring 20 questions. In total, 103 cardiologists (from 92 Italian hospitals) answered the survey. Vascular access during CIED implants was considered the most complex step following lead placement by 54 (52.4%) respondents and the most complex for 35 (33.9%). In total, 54 (52.4%) and 49 (47.6%) respondents considered the cephalic and subclavian vein the first option, respectively (intrathoracic and extrathoracic subclavian/axillary vein by 22 and 27, respectively). In total, 45 (43.7%) respondents performed close arterial femoral accesses manually; only 12 (11.7%) respondents made extensive use of vascular closure devices. A total of 46 out of 103 respondents had experience in ultrasound-guided vascular accesses, but only 10 (22%) used it for more than 50% of the accesses. In total, 81 (78.6%) respondents wanted to increase their ultrasound-guided vascular access skills. Reducing complications is a goal to reach in cardiac stimulation and electrophysiological procedures. Our survey shows the heterogeneity of the vascular approaches used in Italian centres. Some vascular accesses were proved to be superior to others in terms of complications, with ultrasound-guided puncture as an emerging technique. More effort to produce the standardization of vascular accesses could be made by scientific societies.

## 1. Introduction

Venous accesses in cardiac stimulation and in electrophysiological laboratories represents a fundamental step towards implantation safety and efficacy. Vascular complications during cardiovascular implantable electronic device (CIED) implant are rare, but they could have serious implications. Different venous accesses are currently used for CIED implantations, but each approach presents potential advantages and disadvantages. Cephalic vein access is very safe for the lead, which is the weak element of CIED, but it is not always available. Cephalic vein cutdown could be time-consuming and the size of the vein may not be large enough for the lead. Subclavian vein access is faster and simpler, but prone to lead integrity issues, particularly if the puncture is made on the first rib (intrathoracic portion of the vein). Finally, axillary vein access (i.e., the extrathoracic portion of the subclavian vein) has the advantages of both approaches, but it may require fluoroscopy (and venogram) or ultrasound. Venous and arterial accesses are fundamental steps during electrophysiological (EP) study or ablation. In the last ten years, EP tools have increased in number and size. Therefore, the risk of vascular complications is not negligible, both in terms of frequency and clinical implications. With these assumptions, the purpose of our survey was to evaluate common Italian practice concerning how vascular access maneuvers are perceived by operators and which vessels and techniques are preferentially used.

## 2. Materials and Methods

The present survey was endorsed by the AIAC. All Italian centers with experience in CIED implantation and electrophysiology were invited to participate. From May 2020 to November 2020, each center received an online questionnaire using dedicated survey software (Survey monkey). Data were collected by means of online internet entry. An electronic form was created, on which respondents described their profile (age, number and kind of procedures), vascular access preferences and technical device available in their hospital. The survey consisted of a total of 20 questions, reported in the Supplementary Material.

Approval from the ethics committee was not required as the data were derived from a survey of cardiologists from different centers and did not involve patients.

## 3. Results

### 3.1. Respondents’ Characteristics

**Figure 1:**
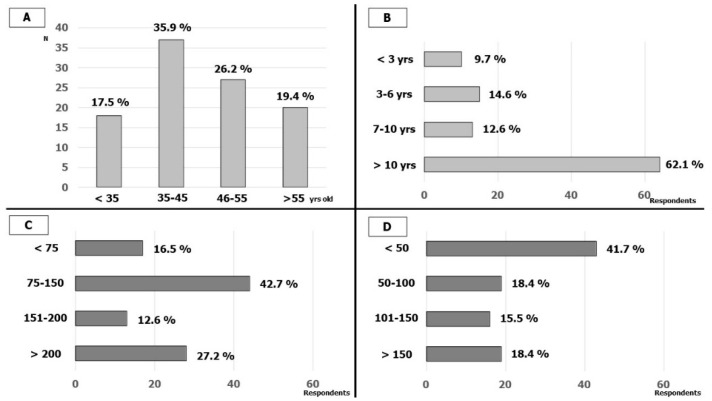
Respondents’ characteristics: (**A**) Age of the respondents; (**B**) respondents’ experience in EP-LAB; (**C**) number of CIED implantations by year; (**D**) number of EP studies and ablations by year.

One-hundred-and-three cardiologists (from 92 Italian hospitals, about 1/3 of AIAC-affiliated hospitals that perform CIED implantation and EP) answered the survey. In total, 18 (17.5%) were less than 35 years old, 37 (35.9%) were between 35 and 45 years old, 27 (26.2%) were between 46 and 55 years old and 20 (19.4%) were more than 55 years old. In total, 64 (62%) respondents had long experience in EP lab (more than 10 years) while only 10 (9.7%) had less than 3 years. The cardiologists interviewed were predominantly experienced in cardiac stimulation and 15 (15%) also performed procedures in pediatric populations; in detail, 86 (84.5%) had an annual number of implants of more than 75 CIED (41 more than 150). The annual number of electrophysiological procedures (EP procedures) was lower among the participants; 48 (46.6%) respondents performed fewer than 50 EP procedures per year and only 19 (18.4%) performed more than 150. The characteristics of the respondents are reported in Figure 1.

### 3.2. Vascular Accesses in Cardiac Stimulation

Vascular accesses did not represent a worrying step for the CIED implanters; on a scale from 0 (no concern) to 10 (very much concern), 51 (49.6%) answered less than 4 and only 7 (6.8%) more than 7.

By dividing the CIED implant procedure into four steps (vascular access, lead placement, pocket surgery and sutures), vascular access was still considered the most technically complex step following lead placement by 54 (52.4%) respondents and the most complex for 35 (33.9%). There was no relationship between the age of the operator/respondent and the reported order of complexity of the different steps.

**Figure 2:**
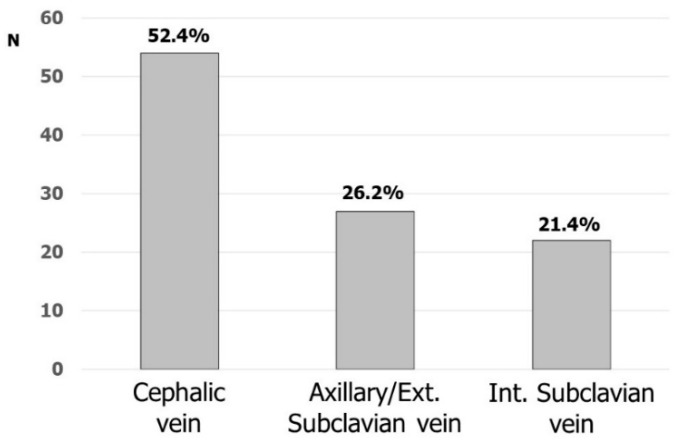
Favorite venous accesses for CIED implantation.

Fifty-four (52.4%) and forty-nine (47.6%) respondents considered cephalic and subclavian vein the first option, respectively (intrathoracic and extrathoracic subclavian/axillary vein by 22 and 27, respectively). Figure 2 shows the favorite accesses for CIED implantation. Cephalic vein cutdown was the most commonly used vascular access: 45 (43.7%) participants were used to placing at least one lead in this vein and, most of the time up to two leads, in more than 80% of their implantations. Nineteen respondents were used to directly approaching the axillary/subclavian vein puncture without searching the cephalic vein, while sixty-seven (65.0%) respondents were used to puncturing the subclavian/axillary vein after the skin incision. The remaining respondents were used to puncturing the subclavian/axillary vein before the skin incision.

### 3.3. Vascular Accesses in Electrophysiology

Vascular accesses during EP procedures were not considered the most worrying step but the concern of the operators was greater than for vascular accesses during CIED implants. On a scale from 0 (no concern) to 10 (very much concern), 50 (48.5%) answered more than 4 (only 1 answered more than 8). In total, 101 out of 103 respondents were used to performing vascular accesses on their own; 18 (17.4%) pointed out that sometimes an interventional cardiologist helped them with arterial or difficult venous accesses. Thirty (29.1%) respondents declared that they punctured the common femoral vein, while the remaining respondents punctured further downstream from the inguinal root.

The closure of the arterial femoral accesses was always performed manually by 45 (43.7%) respondents, whereas mechanical devices were sometimes preferred (44.7%); only 12 (11.7%) respondents made extensive use of these devices.

### 3.4. Ultrasound-Guided Vascular Access

Sixty-six (64.1%) respondents claimed to have an ultrasound system with a vascular probe available in the EP laboratory, whereas 26 respondents claimed to have it only on demand, and 11 do not have one at all.

In total, 57 respondents never used ultrasound to find vascular accesses.

Among 46 respondents that had used ultrasound for the vascular accesses, 29 (63 %) used it for fewer than 20% of accesses, 7 (15%) for between 20–50% of the accesses, and 10 (22%) for more than 50% of the accesses. A total of 11 (24%) respondents always used echography during CIED implantation while the remaining respondents only did so after at least three failed blind puncture attempts.

**Figure 3:**
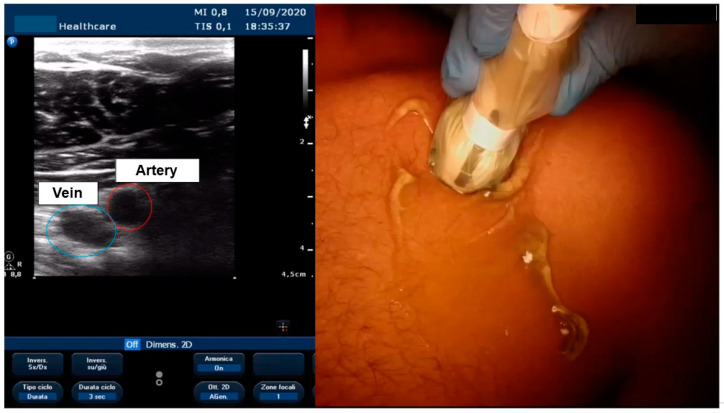
Visualization of axillary vein with echo.

Figure 3 and Video S1 report echo-guided vascular access and vein puncture.

During EP interventions, 10 (22%) used echo guidance only before the puncture to view the anatomy, 26 (56%) used it during the puncture, and 10 (22%) also used it to confirm that the guides were in the vessels.

Eighty-one (78.6%) respondents said that they wanted to start using echo or increase its use for vascular accesses, and 77 (74.8) would be interested in a vascular access course.

## 4. Discussion

The European Heart Rhythm Association (EHRA) recently published an expert consensus statement regarding the optimal implantation technique for pacemakers and cardioverter defibrillators [1]. In this statement, an extensive review of vascular access techniques and outcomes was performed. Reducing CIED-related adverse events and enhancing lead longevity are desirable effects described in the document.

This survey was conducted before the publication of the EHRA consensus statement and aimed to describe current Italian implantation practice while underlining possible weak points that could be targeted by future courses. We also investigated common practice regarding vascular access during electrophysiological procedures and the use of vascular closure devices.

Vascular access does not worry the operator even though it still represents one of the most complex aspects of CIED implantation. This consideration by the respondents probably derives from the fact that most of them had significant experience in CIED implantation; therefore, even though vascular access is a technically complex step in implantation, it does not generate major concerns.

The survey shows that a cephalic vein approach was routinely attempted by half of the respondents. These results are similar to the EHRA survey, which reported that the cephalic vein is the first approach in 60% of centres [2]. Compared to the subclavian, the cephalic vein approach has been proven to reduce complications in several meta-analyses and studies; in particular, it has reduced pneumothorax (PNX) and lead-related issues, such as conductor fracture and insulation defects [2,3,4]. Its disadvantages are its longer procedure, higher blood loss, and lower success rate (of approximately 70%); the latter could be improved to 90% by hydrophilic guide use [5,6]. Axillary/extrathoracic subclavian vein puncture offers a valid alternative to cephalic vein cutdown in terms of lead longevity; it avoids soft-tissue entrapment, which causes the “subclavian crushing syndrome” [4,7]. This vascular approach seems to reduce PNX but may require contrast-guided puncture or a 30–35° caudal fluoroscopy view [8,9]. Ultrasound-guided (US-guided) puncture seems to speed up vascular access with a rate of complications comparable to cephalic vein cutdown and with low operator skill dependency [10]. Furthermore, it reduces potential radiation-related damage (which can be deterministic or stochastic) and avoids contrast medium use with potential kidney injury. Several respondents have access to an ultrasound with a vascular probe, but US-guided puncture is still underused; the majority of respondents would like to attend a US vascular course to improve their skills.

Despite this desire, it should be noted there are studies showing that even operators with no previous experience in US-guided axillary vein puncture can start performing the procedure because the learning curve of this approach is short, and the results are excellent, even with self-learning [11,12].

Almost all the respondents achieved vascular access on their own during electrophysiological procedures, but few of them used vascular closure devices (VCD) routinely. VCDs have been proven to reduce time to hemostasis and time to mobilization compared to manual compression (MC), with a comparable or inferior number of adverse events. Very few trials compare different types (i.e., anchor/plug-mediated devices, extravascular devices, suture-based devices) of VCD and most of the data come from meta-analyses, registry studies, and systematic reviews [11,12,13,14,15,16,17]. The spread of VCDs could reduce hospital stay after EP procedures [18,19].

### Limitations

As with all surveys, the respondents were self-selected. The replies may therefore not represent the opinions of all the operators in Italy, but only the opinion of 103 of them.

## 5. Conclusions

This survey reports, for the first time, to our knowledge, which types of vascular access are used by operators in cardiac stimulation and electrophysiology and their related criticalities.

The cephalic vein is the most frequently used vascular access for CIED implantation in order to protect the lead as much as possible, even though echo-guided axillary vein puncture is a new type of access that is increasing. Axillary vein access is very safe and makes it possible to protect the lead; its limitation is related to the use of echo guidance, which is not always available in the various cath-laboratories, and operators are not yet experts in this approach. In EP, vascular access was not considered a worrying step, even though the associated complication rate is not negligible, especially for femoral arterial access. Often, emodinamists help electrophysiologists with the access, which underlines the need for dedicated training for vascular accesses in electrophysiology and the use of tools such as echo guidance for puncture and for closing arterial and venous accesses.

## Data Availability

Digital database available from the corresponding author, MZ: matteo.ziacchi@gmail.com.

## Supplementary Materials

The following supporting information can be downloaded at: https://www.mdpi.com/article/10.3390/biology11020265/s1, File S1: Survey AIAC regionale su accessi vascolari; Video S1: Elementi multimediali1.

## Appendix A

* List of AIAC Ricerca Investigators: Basilicata: Andriani A., Ospedale Giovanni Paolo II, Policoro (Matera); Calabria: Infusino T., S. Anna Hospital, Catanzaro; Campania: D’Angelo G., Del Giorno G., Ospedale Maria SS Addolorata, Eboli (Salerno); Stabile G., Clinica Mediterranea, Napoli; D’Onofrio A., Bianchi V., Ospedale Monaldi, Napoli; Sangiuolo R., Ospedale Fatebenefratelli, Napoli; Rapacciuolo A., Azienda Ospedaliera Universitaria Federico II, Napoli; Viciglione C., Ospedale di Marcianise e Piedimonte Matese (Caserta); Emilia Romagna: Sassone B., Virzì S., Ospedale Maria Annunziata, Cento (Ferrara); Bagni E., Scapinelli M., Ospedale di Sassuolo, Sassuolo (Modena); Bandini A., Biancoli S., Ospedale Morgagni Perantoni, Forlì (Forlì-Cesena); Casali E., Policlinico di Modena, Modena; Buia E., Pastori P., Ospedale Di Vaio, Fidenza (Parma); Sabbatani P., Corzani A., Ospedale Bufalini, Cesena (Forlì-Cesena); Tomasi C., Dal Monte A., Giannotti F., Arniani S., Ospedale Santa Maria delle Croci, Ravenna; Zardini M., Placci A., Azienda Ospedaliera, Parma; Biffi M., Martignani C., Diemberger I., Massaro G, Lorenzetti S. Sant’Orsola, Bologna; Piovaccari G., Saporito D., Fabbri F., Ospedale Infermi, Rimini; De Maria E., Ospedale di Carpi (Modena); Bottoni N., Quartieri F., ASMN Reggio Emilia; Bertini M., Malagu M., Ospedale Sant’Anna, Ferrara; Barbato G., Carinci V., Ospedale Maggiore, Bologna; Friuli-Venezia Giulia: Zecchin M., Azienda Sanitaria Universitaria Intergata di Trieste, Trieste; Proclemer A. Azienda Sanitaria Universitaria Integrata di Udine, Udine; Lazio: Azzolini P., FBF Isola Tiberina, Roma; Ammirati F., Santini L. Ospedale G.B. Grassi Ostia Lido, Roma; Pignalberi C., Colivicchi F., Ospedale San Filippo Neri, Roma; Sarli G., Grifoni E., Ospedale S. Sebastiano Martire, Frascati (Roma); Castro A., Iulianella R., Ospedale Sandro Pertini, Roma; Santini M., Gallo S., Aurelia Hospital, Roma; Patruno N., Ospedale S. Giuseppe, Albano Laziale (Roma); Pelargonio G., Perna F., Policlinico Universitario Agostino Gemelli, Roma; Liguria: Laffi M., Rubartelli P., Ospedale Villa Scassi, Genova; Zoni Berisso M., Ospedale Padre A Micone, Genova; Lombardia: De Ponti R., Caravati F., Cardiologia 1 Ospedale di Circolo-University of Insubria Varese; Tondo C., Casella M., Centro Cardiologico Monzino, Milano; Passamonti E., Spotti A., ASST di Cremona, Cremona; Sangiorgio S., Ospedale Fatebenefratelli, Milano; Pani A. ASST Ospedale Manzoni, Lecco; Spaziani D. Ospedale di Magenta, Magenta; Reggiani A., Pepi P., ASTT-Mantova, Mantova; Pecora D., Fondazione Poliambulanza, Brescia; Malaspina D., ASST Santi Paolo e Carlo PO San Carlo Borromeo, Milano; Tarricone D., Ospedale San Paolo, Milano; Della Bella P., Mazzone P., San Raffaele, Milano; Locatelli A. ASST Bergamo Est, Seriate; Belotti G., Ospedale di Treviglio- ASST di Bergamo Ovest, Treviglio (Bergamo); Perego G.B., Brambilla R., Istituto Auxologico Italiano, Milano; Landolina M., Chieffo E, Ospedale Maggiore di Crema, Crema (Cremona); De Filippo P., Ospedale Papa Giovanni XXII, Bergamo; Marche: Cecconi M., Spagnolo D., Ospedale di Civitanova Marche (Macerata); Capucci A., Guerra F., Luzi M., Cipolleta, L., Ospedali Riuniti, Ancona; Mezzetti M., Ospedale di Urbino, Urbino; Palpacelli C., AV3-Stabilimento, Macerata; Molise: Alfieri T., Ospedale S. Timoteo, Termoli (Campobasso); Piemonte: Amellone C., Ospedale Maria Vittoria, Torino; Occhetta E., Dell’Era G., Porcelli S., Ospedale Maggiore della Carità, Novara; Puglia: Marino E., Amico A.F., Ospedale San Giuseppe Da Copertino, Copertino (Lecce); Pellegrino P.L., Ziccardi L., Ospedali Riuniti, Foggia; Potenza D., Ospedale Casa Sollievo della Sofferenza, San Giovanni Rotondo (Foggia); Scianaro M.C., Ignone G., Ospedale Antonio Perrino, Brindisi; Gianfrancesco D., Cannone M., Ospedale Bonomo, Andria (BAT); Pisanò E.C.L., Ospedale Vito Fazzi, Lecce; Accogli M., Ospedale Cardinale G. Panico, Tricase (Lecce); Tunzi F., Piccinni G.C., Ospedale Sacro Cuore di Gesù, Gallipoli (Lecce); Giaccari R., Ospedale Camberlingo, Francavilla Fontana (Brindisi); Rodio G., Russo V., Ospedale SS Annunziata, Taranto; Rillo M., CdC Villa Verde, Taranto; Caldarola P., Resta M., Ospedale San Paolo, Bari; Bonfantino V., Valecce R., Ospedale Di Venere, Bari; D’Agostino C., Carretta D., Cardiologia Ospedaliera Policlinico di Bari, Bari; Marsano P., Ospedale Ignazio Veris, Scorrano (Lecce); Favale S., Luzzi G., Cardiologia Universitaria Policlinico di Bari, Bari; Sai R., Ospedale Francesco Ferrari, Casarano (Lecce); Sardegna: Ocello S., Ospedale SS Carità, Cagliari; Dettori F., Ospedale Civile San Martino, Oristano; Sicilia: Calvi V., Platania F., Policlinico Vittorio Emanuele, Catania; Toscana: Notaristefano P., Ospedale S. Donato, Arezzo; Bongiorni M.G., Paperini L., Azienda Universitaria Ospedaliera, Pisa; Piacenti M., Fondazione Toscana G. Monasterio, Pisa; Umbria: Zingarini, G., Ospedale S. Maria Della Misericordia, Perugia; Veneto: Bertaglia E., Migliore F., Università di Padova, Padova; Molon G., Ospedale Sacro Cuore, Negrar (Verona); Zanotto G., Ospedale Mater Salutis, Legnago (Verona).
