# Supplementary material for: Vascular Accesses in Cardiac Stimulation and Electrophysiology: An Italian Survey Promoted by AIAC (Italian Association of Arrhythmias and Cardiac Pacing)
Source: Biology (Basel). 2022 Feb 8;11(2):265. doi: 10.3390/biology11020265 (PMC8869488; doi:10.3390/biology11020265)
Supplement: Supplementary file 1 [file biology-11-00265-s001.zip › biology-1553051-supplementary/Survey AIAC regionale su accessi vascolari.zip!/Survey AIAC regionale su accessi vascolari.pdf]

SURVEY AIAC EMILIA ROMAGNA 2020:

VASCULAR ACCESS IN ELECTROPHYSIOLOGY AND CARDIAC ELECTRONIC DEVICE IMPLANTATIONS.

Respontent's data

Date of birth:

How long (years) have you been doing electrophysiogy and CIED implant procedures?

1) <3
2) 3>6
3) 6>10
4) >10

How many CIED implants do you do yearly?

1) <75
2) 75<150
3) 150<200
4) >200

How many electrophysiological procedures do you do yearly?

1) <50
2) 50<100
3) 100<150
4) >200

Echo-guided vascular access

Is there an ultrasound system with vascular probe in your EP-lab?

1) yes
2) on-demand only
3) no

Do you use ultrasound-guided vascular access?

1) Yes, but only for femoral vessels
2) Yes, but only for subclavian/axillary vessels
3) Always
4) Never

Ultrasound-guided puncture is used:

1) Always
2) After 3 failed attempts of blind puncture
3) After, at least, 5 failed attempts of blind puncture
4) Never

If you are using an ultrasound for vascular access, which is the percentage of its use?

1) Less than 20% of cases
2) between 20% and 50% of cases
3) Between 50% and 80% of cases
4) More than 80% of cases

CIED implantation procedures

What degree of concern do you attribute to vascular access? (0 low, 10 very much)

0 1 2 3 4 5 6 7 8 9 10

Sort the steps of the procedure in ascending order of complexity, form the most complex (1) to the easiest (4)

1) Vascular access …….
2) Lead insertion …….
3) Pocket creation …….
4) Suture …….

Which is your first choice for vascular access during CIED implantation?

1) Cephalic vein
2) Intrathoracic subclavian vein
3) Axillary/extrathoracic subclavian vein

What is the percentage in which you are able to put at least one lead in cephalic vein?

1) More than 80% of cases
2) between 50% and 80% of cases
3) between 20% and 50% of cases
4) less than 20% of cases
5) Never, I usually do not look for the cephalic vein

How many leads, on average, you place through cephalic vein?

1) None
2) 1
3) 2

Subclavian vein puncture is done:

1) before skin incision, particularly if an ultrasound-guided puncture is done
2) always before skin incision
3) after skin incision

Electrophysiology

What degree of concern do you attribute to vascular access? (0 low, 10 very much)

0 1 2 3 4 5 6 7 8 9 10

Arterial femoral access is done:

1) Always by electrophysiologist
2) Usually by electrophysiologist by sometimes with the help of an hemodynamist.
3) Always by hemodynamist

Arterial femoral access closure is done:

1) Always manually
2) Manually most of the time but sometimes with closure devices
3) Usually with closure devices and the sometimes manually
4) Always with closure devices

Venous femoral access is usually done:

1) at the level of the common femoral vein
2) at the level of the femoral vein

Optional questions

Those who use an ultrasound during vascular access they uses it:

1) to evaluate vascular anatomy before a blind puncture
2) for an ultrasound-guided puncture
3) both for an ultrasound-guided puncture and wire position check

Would you be interested in a ultrasound course for vascular access?

1) yes
2) no
